# Complete Genome Sequence of Campylobacter hepaticus Strain UF2019SK1, Isolated from a Commercial Layer Flock in the United States

**DOI:** 10.1128/MRA.01446-20

**Published:** 2021-03-25

**Authors:** Ananta Arukha, Thomas N. Denagamage, Gary Butcher, Subhashinie Kariyawasam

**Affiliations:** a Department of Comparative, Diagnostic, and Population Medicine, University of Florida, Gainesville, Florida, USA; b Department of Large Animal Clinical Sciences, College of Veterinary Medicine, University of Florida, Gainesville, Florida, USA; University of Arizona

## Abstract

The thermophilic *Campylobacter* species Campylobacter hepaticus is the causative agent of spotty liver disease (SLD) in chickens. This announcement describes the complete genome sequence of *C. hepaticus* strain UF2019SK1, isolated from the liver of a commercial layer chicken with SLD in the United States.

## ANNOUNCEMENT

Spotty liver disease (SLD), or spotty liver syndrome, in chickens is an acute disease caused by the thermophilic *Campylobacter* species Campylobacter hepaticus (1–5). The disease is characterized by 1- to 2-mm grayish-white or cream spots in the liver, increased mortality, and a drop in egg production (3, 5, 6). Although cases of SLD have been reported most commonly in free-range and barn-housed commercial layer flocks around the time of peak production, occasional outbreaks have occurred in caged layers, as well as in broiler and layer breeders (1, 4, 5, 7). With the steady increase in free-range commercial layer management practices worldwide, *C. hepaticus* may remain as a major bacterial pathogen of table egg production. Although several genome sequences of *C. hepaticus* strains from other countries have been deposited in public databases (1, 2, 8, 9), to the best of our knowledge, only one *C. hepaticus* strain isolated in the United States has been sequenced to date (GenBank accession number CP063536.1). In the present study, the chromosome of *C. hepaticus* strain UF2019SK1, which was isolated from the liver of a 21-week-old chicken in a caged layer flock in Florida showing the typical signs and lesions of SLD, was sequenced and fully closed. For isolation of *C. hepaticus*, the liver specimen was collected aseptically, transported to the laboratory in Stuart’s transport medium (Oxoid) within 4 h of collection, and processed as described previously (1). Briefly, a section of the liver was macerated in 5 ml of modified Preston broth and incubated for 7 days at 37°C. After 7 days of incubation, subcultures were spread onto 5% sheep blood agar (Remel, Lenexa, KS) and incubated at 37°C for 3 days in a microaerophilic environment (85% N_2_, 7.5% CO_2_, 7.5% O_2_) created with a MicroAero gas generator and a Mitsubishi AnaeroPack rectangular jar (Fisher Scientific, Portsmouth, NH). At this point, Gram staining and an oxidase test were performed to identify presumptive *Campylobacter* colonies (Gram negative and oxidase positive). The suspected *Campylobacter* colonies were grown one more time on 5% sheep blood agar at 37°C in a microaerophilic atmosphere for 3 days to ensure purity. The isolated *Campylobacter* colonies were confirmed as *C. hepaticus* by PCR, which targeted the glycerol kinase gene (*glk*), and 16S rRNA gene sequencing, as described previously (6, 10). Frozen stocks of *C. hepaticus* were made in 1% protease peptone water containing 15% glycerol and stored at −80°C for long-term storage. For DNA extraction, bacteria were grown from frozen stocks on 5% sheep blood agar under microaerophilic conditions at 37°C for 3 days. A single colony of bacteria was suspended in 100 μl phosphate-buffered saline (PBS) before spreading onto 5% sheep blood agar and then incubated as above. The entire bacterial growth was harvested into a tube containing 1 ml of 0.1 M PBS (pH 7.2) solution and subjected to *glk* gene PCR to confirm the bacterial growth as *C. hepaticus* before proceeding to DNA extraction.

Genomic DNA was extracted using a Genomic-tip 100/G kit (Qiagen, Inc., Germantown, MD, USA) according to the manufacturer’s instructions. The DNA sample was quantified using a Qubit version 2.0 fluorometer (Life Technologies, Carlsbad, CA, USA), and the sample purity and integrity were checked using NanoDrop and pulsed-field gel analysis, respectively. DNA library preparations, sequencing reactions, and initial bioinformatics analysis were conducted at Genewiz, LLC (South Plainfield, NJ, USA). An ∼10-kb library for PacBio Sequel was constructed using the SMRTbell template prep kit version 1.0 (PacBio, Menlo Park, CA, USA). The library was bound to polymerase using the Sequel binding kit version 2.0 (PacBio) and loaded onto a PacBio Sequel instrument using the MagBead kit version 2 (PacBio). Sequencing was performed on a single PacBio Sequel single-molecule real-time (SMRT) cell, using Instrument Control Software version 5.0.0.6235, Primary analysis software version 5.0.0.6236, and SMRT Link version 5.0.0.6792. Reads were assembled using HGAP 4 (within the SMRT Link suite) and Canu. Sequencing yielded a total of 45,335 corrected PacBio reads with a read distribution (*N*_50_) of 5,346 bp and a total of 1,600,012,565 bp. At this point, the corrected sequences were assembled with the CLC Genomics Workbench software version 20 (Qiagen, Inc.). The initial assembly was performed with reference-guided assembly using *C. hepaticus* strain HV10 (GenBank accession number NZ_CP031611) as the reference. *De novo* assembly was used to complete and verify the assembled genome sequence. After obtaining a draft circular genome sequence, the remaining gaps were filled by a primer-walking approach. At this point, the circularized genome sequence of *C. hepaticus* was annotated using NCBI Prokaryotic Genome Annotation Pipeline (PGAP; https://www.ncbi.nlm.nih.gov/genome/annotation_prok/) (11) and was independently analyzed on the Rapid Annotations using Subsystems Technology (RAST) server (http://rast.nmpdr.org/). (12). Since there were no inconsistencies observed, the PGAP annotation was used as the public-facing version. The SEED viewer (http://pubseed.theseed.org/) (13) was used for subsystem functional categorization of the predicted open reading frames (ORFs) obtained from annotation and initial subsystem assignments with RAST. Default parameters were used for all software, including those used for genome assembly and annotation, unless otherwise specified.

Analysis of the *C. hepaticus* UF2019SK1 chromosome revealed that it consists of a circular chromosome of 1.52 Mb. The sequence mapped to 1,525 open reading frames (ORFs), which included 1,470 coding DNA sequences (CDS), 9 rRNAs, 43 tRNAs, and 3 noncoding RNAs. Similar to other sequenced *C. hepaticus* strains, the average G+C content of the UF2019SK chromosome was approximately 28%. The SEED viewer assigned about 33% of genes from the predicted ORFs from RAST annotation to a particular biochemical pathway or a subsystem wherein protein metabolism and amino acid utilization pathways made up the two largest subsystems. The genomic data reported in this announcement will be useful in future studies of *C. hepaticus* pathogenesis and vaccine development.

### Data availability.

The complete annotated chromosome was deposited in NCBI GenBank under accession number CP065357.1, BioProject accession number PRJNA681575, and BioSample accession number SAMN16960974. The raw reads were deposited in the NCBI SRA under accession number SRX10000234.
